# Long-Term Follow-Up of COVID-19 Convalescents—Immune Response Associated with Reinfection Rate and Symptoms

**DOI:** 10.3390/v15102100

**Published:** 2023-10-17

**Authors:** Anna Seller, Christopher Hackenbruch, Juliane S. Walz, Annika Nelde, Jonas S. Heitmann

**Affiliations:** 1 Clinical Collaboration Unit Translational Immunology, German Cancer Consortium (DKTK), Department of Internal Medicine, University Hospital Tuebingen, Otfried-Müller-Str. 10, 72076 Tuebingen, Germany; 2 Department of Obstetrics and Gynecology, University Hospital Tuebingen, Calwerstraße 7, 72076 Tuebingen, Germany; 3 Department of Peptide-Based Immunotherapy, Institute of Immunology, University Hospital Tübingen, Otfried-Müller-Str. 10, 72076 Tuebingen, Germany; 4 Cluster of Excellence iFIT (EXC2180) “Image-Guided and Functionally Instructed Tumor Therapies”, University of Tübingen, Röntgenweg 11, 72076 Tuebingen, Germany

**Keywords:** SARS-CoV-2, COVID-19, post-COVID syndrome, immune response

## Abstract

SARS-CoV-2 has spread worldwide, causing millions of deaths and leaving a significant proportion of people with long-term sequelae of COVID-19 (“post-COVID syndrome”). Whereas the precise mechanism of post-COVID syndrome is still unknown, the immune response after the first infection may play a role. Here, we performed a long-term follow-up analysis of 110 COVID-19 convalescents, analyzing the first SARS-CoV-2-directed immune response, vaccination status, long-term symptoms (approximately 2.5 years after first infection), and reinfections. A total of 96% of convalescents were vaccinated at least once against SARS-CoV-2 after their first infection. A reinfection rate of 47% was observed, and lower levels of anti-spike IgG antibodies after the first infection were shown to associate with reinfection. While T-cell responses could not be clearly associated with persistent postinfectious symptoms, convalescents with long-term symptoms showed elevated SARS-CoV-2-specific antibody levels at the first infection. Evaluating the immune response after the first infection might be a useful tool for identifying individuals with increased risk for re-infections and long-term symptoms.

## 1. Introduction

Severe acute respiratory syndrome coronavirus 2 (SARS-CoV-2) causing coronavirus disease 2019 (COVID-19) has spread worldwide, with more than 750 million documented cases and nearly 7 million deaths to date [1]. The introduction of effective SARS-CoV-2 vaccines largely reduced the number of life-threatening COVID-19 cases, which resulted in the suspension of restrictions to daily life and even in the health care system [2].

Usually, infected individuals present with a variety of disease symptoms, including fever, fatigue, loss of smell and taste, headache, cough, and/or shortness of breath [3]. Of note, even in less severe cases, numerous COVID-19 convalescents suffer from long-term sequelae (“post-COVID syndrome”) that may result in significant limitations in daily life. Moreover, there are profound socioeconomic consequences due to prolonged work absences; thus, a significant loss of labor force may occur [2,4,5,6,7,8,9,10,11,12]. As the potential risk for post-COVID syndrome—severe course of COVID-19—was identified, the risk is lower in convalescents after asymptomatic to moderate COVID-19 [4,7,11,12].

After symptoms resolve, patients are usually considered to be protected from reinfection for several months by the immunological memory of the humoral and cellular immune system [13]. The humoral immune response, i.e., anti-SARS-CoV-2 antibody levels, has been reported to be increased in patients with more severe and acute COVID-19 [14]. As was recently shown, increased SARS-CoV-2-specific antibody levels were also observed in individuals with post-COVID syndrome in comparison to both asymptomatic and mildly affected convalescents [15]. In acute COVID-19 patients, SARS-CoV-2-directed T-cell responses were—in contrast to antibody responses—not elevated in severe COVID-19 cases, but equally strong for different disease severities, highlighting the strength of the T-cell-based immunity for viral clearance [16].

In this study, we performed a long-term follow-up of 110 COVID-19 convalescents from 2020 [17]. We analyzed the occurrence of different post-infectious symptoms (2.5 years after the first positive PCR result), as well as reinfection rates, vaccination status, and the association with the humoral and cellular immune response against SARS-CoV-2, which had been assessed shortly after the first infection.

## 2. Materials and Methods

### 2.1. Convalescents and Blood Samples

Blood and serum samples from convalescent volunteers after asymptomatic and mild or moderate symptomatic SARS-CoV-2 infection according to WHO criteria (n = 110) were collected at the University Hospital Tübingen in April 2020 [18]. The questionnaire-based follow-up assessment of donor characteristics, vaccinations, long-term symptoms and additional infections was performed approximately 2.5 years after the first infection. Informed consent was obtained in accordance with the Declaration of Helsinki protocol. The study was approved by and performed according to the guidelines of local ethics committees (179/2020/BO2). The sample collection in terms of peripheral blood mononuclear cells (PBMCs) and serum was performed approximately 3–8 weeks after the end of symptoms and/or the negative virus smear. Data on the existence of post-COVID syndrome were retrieved five to six months after PCR-confirmed first SARS-CoV-2 infection from a previous publication [15]. Symptom categories were determined by subjective disease symptoms (none, mild, moderate, and severe, as reported by the questionnaire) of individual donors. Detailed donor characteristics and the distribution of symptoms, respectively, as well as their severity, are provided in Table 1 and Table S1. Data on antibody levels, T-cell responses, and post-infectious symptoms were retrieved from previous publications [15,16,17,19].

### 2.2. Follow-Up Assessment of Convalescents

The questionnaire-based assessment of vaccinations (yes/no—if yes, how often; long-term sequelae after vaccination), long-term post-infectious symptoms and grading of severity (none, mild, moderate, and severe), as well as the assessment of second or third SARS-CoV-2 infections, was performed approximately 2.5 years after the first infection. The following symptoms were assessed: breathlessness, fatigue, difficulty concentrating, reduced performance, sleep disorders, depression, loss of taste/smell, muscle/joint pain, hearing loss/disorder, headache, menstruation disorders, and others. Questions concerning second or third SARS-CoV-2-infections focused on timing from the first infection, hospitalization, medication, symptoms, and course compared to the first infection. The severity of symptoms was assessed by self-reported grading (grading for questions: none, mild, moderate, and severe).

### 2.3. T-Cell and Antibody Responses

Data on SARS-CoV-2-specific T-cell responses were assessed by an interferon-γ (IFN-γ) enzyme-linked immunospot (ELISpot) assay against the previously described SARS-CoV-2-specific and cross-reactive epitope compositions for the human leukocyte antigen (HLA) class I and HLA-DR. The SARS-CoV-2 epitope compositions were designed from immunogenic SARS-CoV-2-derived T-cell epitopes, which were derived from different open reading frames, including spike, nucleocapsid, and membrane proteins. The SARS-CoV-2-specific composition was recognized exclusively in convalescent patients after SARS-CoV-2 infection and not in SARS-CoV-2-unexposed individuals. In contrast, the SARS-CoV-2-cross-reactive composition was recognized in convalescent patients after SARS-CoV-2 infection and in SARS-CoV-2-unexposed individuals, most likely due to the cross-reactivity with common coronaviruses (HCoV-OC43, HCoV-229E, HCoV-NL63, and HCoV-HKU1). The HLA class I and HLA-DR epitope compositions cover several different HLA class I and HLA-DR allotypes, respectively, to allow for the standardized evaluation and determination of intensities of SARS-CoV-2-specific T-cell responses [17]. The intensity of T-cell responses was measured as mean spot counts of duplicates in the ELISpot assay normalized to 5 × 10^5^ cells minus the normalized mean spot count of the respective negative control. Data on anti-SARS-CoV-2 nucleocapsid antibody index values (including IgG and IgA) were assessed by the Elecsys^®^ anti-SARS-CoV-2 immunoassay (Roche Diagnostics, Basel, Switzerland), and anti-SARS-CoV-2-spike IgG-antibody levels were assessed by Euroline Anti-SARS-CoV-2^®^ assay (Euroimmune, Luebeck, Germany). The latter assay demonstrated borderline cross-reactivity in 7% of samples from patients with a common-cold coronavirus infection (NL63 and OC43) [20]. For the Elecsys^®^ anti-SARS-CoV-2 immunoassay, no cross-reactivity was observed for the common-cold coronaviruses (229E, NL63, OC43, and HKU1), and the specificity of this assay is 99.5% [21].

Data on T-cell responses and antibody levels 5–6 weeks after the first infection as well as post-infectious symptoms 5–6 months after the first infection were retrieved from previous publications [16,17,19,22].

### 2.4. Software and Statistical Analysis

Data are displayed as a median with range, box plots with a median and 25th and 75th quartiles, min/max whiskers, and individual data points. Continuous data were tested for distribution and individual groups were tested by the use of an unpaired Mann–Whitney U-test or Kruskal–Wallis test. Missing data were included in tables and the descriptive analysis. Statistical analyses were conducted using JMP Pro (SAS Institute, v.16, Carry, NC, USA) software. *p*-values < 0.05 were considered significant. Graphs were plotted using GraphPad Prism v.9.4.1.

## 3. Results

### 3.1. Clinical Characteristics of COVID-19 Convalescents

For this study, we analyzed long-term symptoms 2.5 years after first SARS-CoV-2 infection and their association with the SARS-CoV-2-specific antibody and T-cell responses after first infection of 110 convalescent donors with an asymptomatic to moderate COVID-19 course. Furthermore, data on vaccinations against SARS-CoV-2 and reinfections were collected. The median age of convalescents at the time of first infection was 49 (range 20–75) years, with an equal gender distribution (female:male ratio = 1.08:1) (Table S1). None of the donors were hospitalized, had severe/life-threatening symptoms according to the WHO criteria [18], or were vaccinated prior to the first infection. Antibody and T-cell responses were assessed 16–59 days (median 41 days) after the first positive PCR [5].

### 3.2. Prevalence of Long-Term Symptoms and Severity

Overall, 65% of convalescents reported the presence of at least one long-term symptom 2.5 years after the first infection. The most frequently reported symptoms were fatigue (42%) followed by difficulty concentrating and reduced performance (34% each). A total of 30% of participants indicated some level of ongoing breathlessness, 28% reported sleep disorders, 23% reported muscle/joint pain, and 22% reported depression. Headache (20%), loss of taste/smell (13%), and hearing loss/disorder (11%) were experienced less often. In terms of ongoing symptoms, 25%, 25%, and 15% of convalescents reported at least one symptom as mild, moderate, and severe, respectively. Most frequently, severe fatigue was reported (9% of subjects), and 5% reported either severe sleep disorders, difficulty concentrating, or muscle/joint pain. Only 3% of convalescents reported a severe reduced performance and severe loss of taste/smell, followed by 2% with severe hearing loss/disorder, and menstruation disorders experienced by 1% with severe depression, as well as severe headaches, according to the questionnaire. None reported severe breathlessness. All other reported symptoms were mild to moderate.

Clinical data and details regarding the distribution of severity of the respective symptom groups are presented in Table 1.

### 3.3. Vaccinations

Almost all convalescents (96%) were vaccinated against SARS-CoV-2 at least once after their first infection. A total of 43% of donors received two, 41% received three and 6% (7/110) received four vaccine doses (Table 2). The most frequently used vaccine was Tozinameran (“Comirnaty” from BioNTech/Pfizer, 67%), followed in descending frequency by Elasomeran (“Spikevax” by Moderna, 21%), AZD1222 (“Vaxzevria” by AstraZeneca, 9%), Ad26.COV2.S (“Jcovden” by Janssen, 2%), and others (1%). In the vaccinated group, 3% reported symptoms that they themselves considered to be long-term effects of vaccination (Table 2).

### 3.4. Reinfections

We observed a reinfection rate of 47% (Table 3). For the majority of convalescents (98%), reinfection occurred more than one year after the first infection (range 340–987 days, median 798 days. Four subjects were reinfected for a third time within 61–181 days after the second SARS-CoV-2 infection (median 152 days). All four participants with three infections had been vaccinated at least once against SARS-CoV-2, one even four times.

In terms of symptom severity, none of the reinfected subjects was hospitalized. For subsequent infections, symptom severity was mostly perceived as moderate (35% or 50% for second or third infection, respectively) to severe (61% or 50% for second or third infection, respectively) by the individuum (Table S2). Interestingly, when comparing the severity of the second to the first infection, most subjects categorized severity as same or even less. However, about 12% of subjects reported worse symptoms. No SARS-CoV-2-specific treatment was initiated in any subject, but 29% of patients took nonsteroidal anti-inflammatory drugs for symptom relieve.

### 3.5. Antibody Levels and Long-Term COVID-19 Symptoms

**Figure 1:**
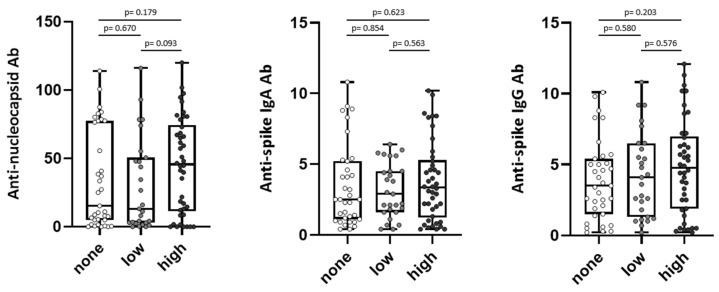
Symptom scores of long-term symptoms and antibody response in SARS-CoV-2 convalescents. Anti-nucleocapsid antibody (Ab) (**left**) and anti-spike Ab levels (IgA **middle**, IgG **right**) were assessed in convalescent donors (n = 110) 5–6 weeks after positive PCR at first infection. Convalescents were grouped into “none” = 0 symptoms (n = 38), “low” = 1–2 symptoms (n = 28), and “high” = 3–10 symptoms (n = 44) at about 2.5 years after first infection. Levels of anti-spike Ab are shown as ratio above threshold value. Levels of anti-nucleocapsid Ab are shown as an index value. Data are presented as box plots with 25th and 75th percentiles and min/max whiskers. *p*-values were calculated by Mann–Whitney U-test. *p*, *p*-value; Ab, antibody.

We compared anti-SARS-CoV-2 antibody levels of convalescent donors after the first infection [17] with the presence of subjective long-term symptoms about 2.5 years after the first infection. To assess SARS-CoV-2-directed humoral immune responses in convalescents with different long-term symptoms, convalescents were grouped according to the number of reported symptoms into three groups: Group one “none” (0 symptoms, n = 38 (35%)), group two with a “low” score for subjective long-term symptoms (1–2 symptoms, n = 28 (25%)), and group three with a “high” score for subjective long-term symptoms (3–10 symptoms, n = 44 (40%)) (Figure 1).

**Figure 2:**
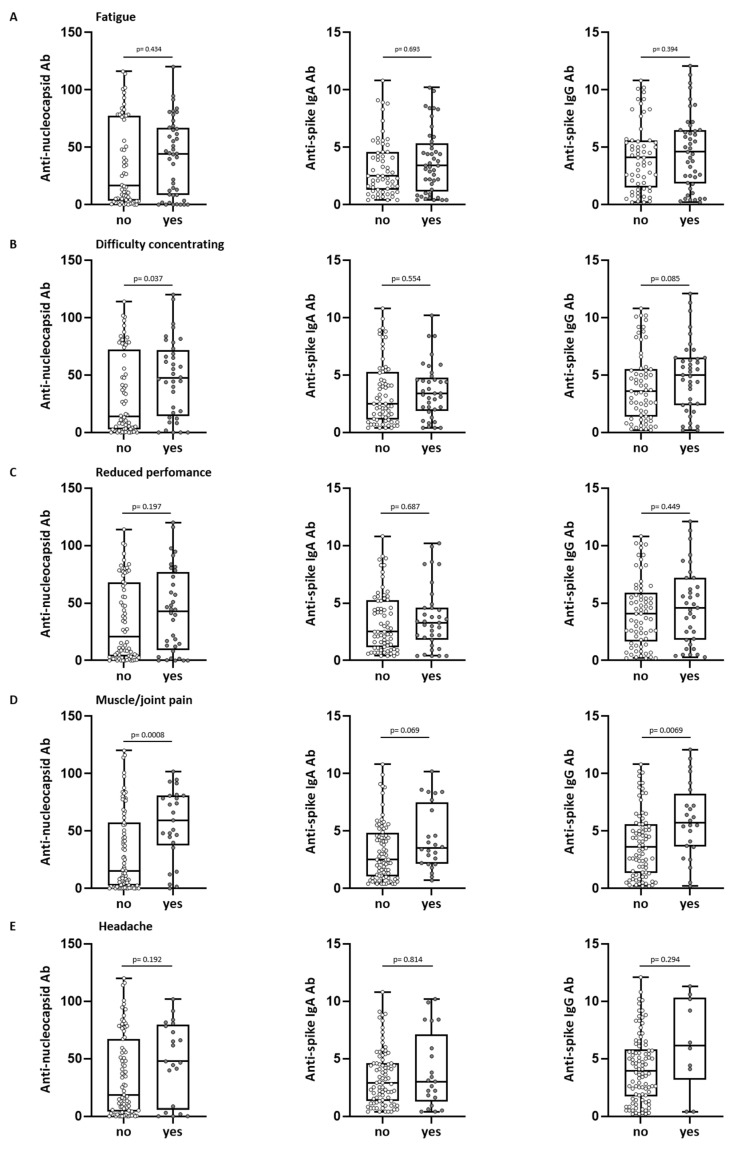
Long-term symptoms and antibody response in SARS-CoV-2 convalescents. Anti-nucleocapsid antibody (Ab) (**left**) and anti-spike Ab levels (IgA **middle**, IgG **right**) were assessed in convalescent donors (n = 110) 5–6 weeks after positive PCR at first infection. Convalescents were grouped into “yes” (reported symptom) or “no” (no perception of symptom) at about 2.5 years after first infection. Shown symptoms are fatigue (**A**), difficulty concentrating (**B**), reduced performance (**C**), muscle and joint pain (**D**) and headache (**E**). Levels of anti-spike Ab are shown as a ratio above the threshold value. Levels of anti-nucleocapsid Ab are shown as an index value. Data are presented as box plots with 25th and 75th percentiles and min/max whiskers. *p*-values were calculated by Mann–Whitney U-test. *p*, *p*-value; Ab, antibody.

With regards to single symptoms, there was a trend towards higher anti-spike IgG levels in convalescents with symptoms of fatigue, difficulty concentrating, reduced performance, muscle and joint pain, headache, breathlessness, sleep disorder, depression, loss of taste/smell, and hearing loss/disorder 2.5 years after first infection, which was significant for symptoms of difficulty concentrating and muscle and joint pain (Figure 2A–E and Figure S1A–E). Anti-nucleocapsid antibody levels were significantly higher (*p* = 0.037) in convalescents, who still reported difficulty concentrating 2.5 years after the first infection (Figure 2B). Subjects reporting persistent muscle and joint pain had significantly higher anti-nucleocapsid antibody levels (*p* = 0.0008) as well as significantly higher anti-spike IgG antibody levels (*p* = 0.0069) after the first infection (Figure 2D). Analyzing the severity of “muscle and joint pain”, we observed significantly higher antibody levels in patients reporting more severe symptoms (anti-nucleocapsid antibody levels *p* = 0.014, anti-spike IgA antibody levels *p* = 0.047, and anti-spike IgG antibody levels *p* = 0.005, Figure S2).

Convalescents with ongoing long-term symptoms displayed a tendency towards increased anti-nucleocapsid and anti-spike IgG antibody levels five to six weeks after the first infection, whereas for anti-spike IgA, no trend was observed (Figure 1).

### 3.6. Antibody Levels and Reinfections

**Figure 3:**
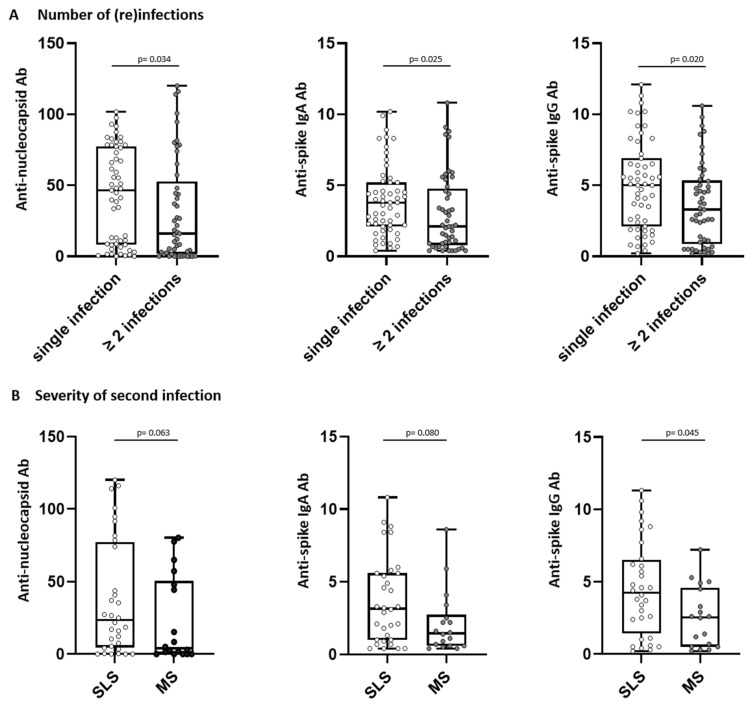
Number of (re)infections (**A**) and severity of second infection (**B**) correlate with antibody response in SARS-CoV-2 convalescents. Anti-nucleocapsid antibody (Ab) (**left**) and anti-spike Ab levels (IgA **middle**, IgG **right**) were assessed in convalescent donors (n = 110) 5–6 weeks after positive PCR at first infection. Convalescents were grouped (**A**) into “single infection” or “≥ 2 infections” and (**B**) according to severity of second infection into similar or less severe (SLS) or more severe course (MS) of second infection as compared to first infection. Levels of anti-spike Ab are shown as ratio above threshold value. Levels of anti-nucleocapsid Ab are shown as an index value. Data are presented as box plots with 25th and 75th percentiles and min/max whiskers. *p*-values were calculated by Mann–Whitney U-test. *p*, *p*-value; Ab, antibody.

Subjects without reinfection showed significantly higher antibody level after the first infection compared to donors with reinfection (anti-nucleocapsid antibody levels *p* = 0.034, anti-spike IgA antibody levels *p* = 0.025, and anti-spike IgG antibody levels *p* = 0.020, Figure 3A). When analyzing the severity of consecutive SARS-CoV-2 infections, convalescents were grouped according to the severity of the second infection into similar or less severe (SLS) and more severe (MS) courses of the second infection compared to the first infection. A comparison between SLS and MS showed that subjects with a similar or less severe course of consecutive infections presented with significantly higher anti-spike IgG levels after the first infection compared to subjects with more severe disease (*p* = 0.045, Figure 3B).

Taken together, initial anti-SARS-CoV-2 antibody levels were significantly lower in individuals who had subsequent reinfections and in individuals with a lower perceived severity of consecutive SARS-CoV-2 infections.

### 3.7. T-Cell Immunity in Association to Long-Term COVID-19 Symptoms and Further Infections

Next, we compared the presence of subjective long-term symptoms with the intensity of SARS-CoV-2-specific and cross-reactive T-cell responses for HLA class I- and HLA-DR-restricted T-cell epitopes [17].

**Figure 4:**
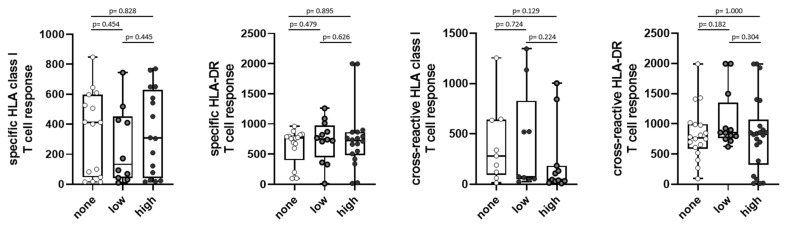
Symptom scores of long-term symptoms with first T-cell response in SARS-CoV-2 convalescents. Intensity of SARS-CoV-2-specific HLA class I (n = 41, first graph) /HLA-DR (n = 47, second graph); T-cell response as well as intensity of SARS-CoV-2-cross-reactive HLA class I (n = 28, third graph)/HLA-DR (n = 52, fourth graph); T-cell response were assessed in convalescent donors 5–6 weeks after positive PCR at first infection. Convalescents were grouped into “none” = 0 symptoms, “low” = 1–2 symptoms, and “high” = 3–10 symptoms at about 2.5 years after first infection. Intensity of T cell responses are shown as mean spot counts of duplicates in the ELISpot assay normalized to 5 × 10^5^ cells minus the normalized mean spot count of the respective negative control. Data are presented as box plots with 25th and 75th percentiles and min/max whiskers. *p*-values were calculated by Mann–Whitney U-test. *p*, *p*-value.

**Figure 5:**
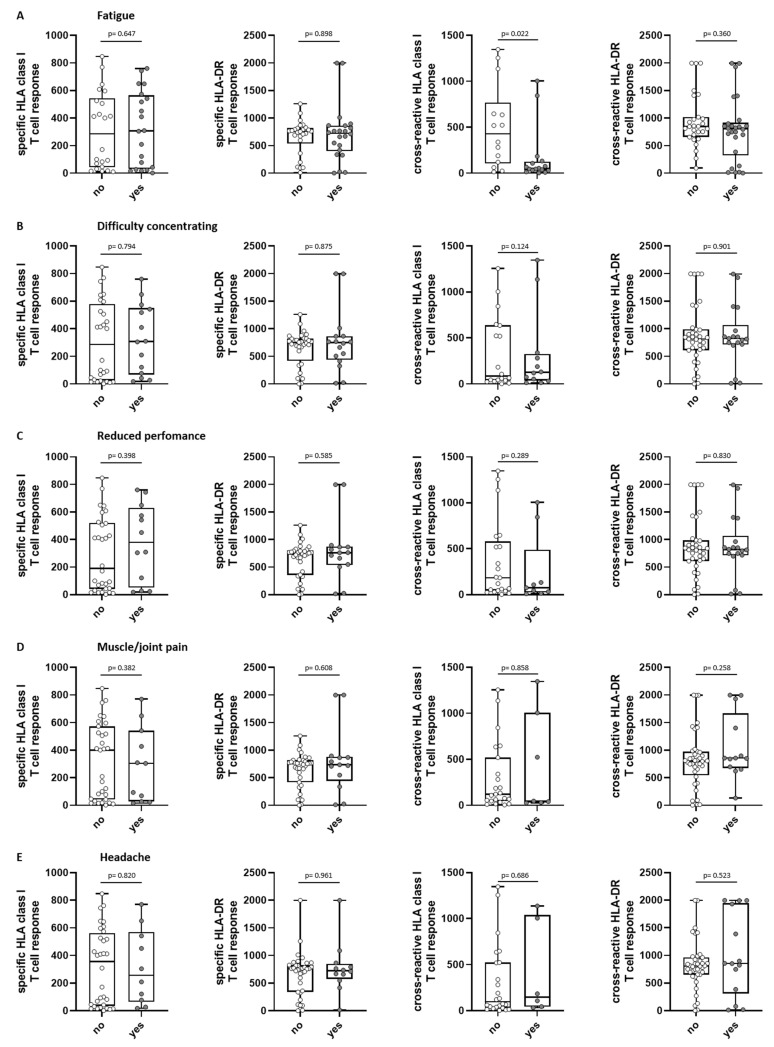
Association of long-term symptoms with initial T-cell response in SARS-CoV-2 convalescents. Intensity of SARS-CoV-2-specific HLA class I (n = 41, first graph)/HLA-DR (n = 47, second graph); T-cell response as well as intensity of SARS-CoV-2-cross-reactive HLA class I (n = 28, third graph)/HLA-DR (n = 52, fourth graph); T-cell responses were assessed in convalescent donors 5–6 weeks after positive PCR at first infection. Convalescents were grouped into “yes” (reported long-term symptom) or “no” (no perception of symptom) at about 2.5 years after first infection. Shown symptoms are fatigue (**A**), difficulty concentrating (**B**), reduced performance (**C**), muscle and joint pain (**D**) and headache (**E**). Intensity of T-cell responses are shown as mean spot counts of duplicates in the ELISpot assay normalized to 5 × 10^5^ cells minus the normalized mean spot count of the respective negative control. Data are presented as box plots with 25th and 75th percentiles and min/max whiskers. *p*-values were calculated by Mann–Whitney U-test. *p*, *p*-value.

The intensity of SARS-CoV-2-specific and cross-reactive T-cell responses against the HLA class I and HLA-DR epitope compositions did not differ significantly for the different symptom scores (Figure 4). When having a closer look at the individual symptoms, the presence of the symptom “fatigue” was associated with a significantly lower cross-reactive T-cell response for the HLA class I epitope composition (*p* = 0.022, Figure 5A). For the other symptoms, no statistical significance was reached (Figure 5B–E).

Subjects without reinfection showed a tendency towards a lower SARS-CoV-2-specific HLA class I-directed T-cell response than those who suffered further infections in the course, whereas no difference could be observed for the other epitope compositions (Figure S3).

In summary, no association of the initial SARS-CoV-2-directed T-cell response intensity with reinfection was observed (Figure 5).

### 3.8. Post-COVID Syndrome after 5–6 Months and Long-Term Symptoms at 2.5 Years after First Infection

For further analysis, long-term symptoms for 2.5 years after first infection were compared with previous data on symptoms of the same convalescents 5–6 months after the first infection. Here, the convalescents were grouped into “post-COVID syndrome” and “no post-COVID syndrome”. Based on this grouping, we performed an analysis to observe whether participants suffering from post-COVID syndrome back then were still affected. The long-term symptoms at 2.5 years were grouped according to the number of reported symptoms into three groups. Group one “none” (0 symptoms, n = 38), group two with a “low” score for subjective long-term symptoms (1–2 symptoms, n = 28), and group three with a “high” score for subjective long-term symptoms (3–10 symptoms, n = 44).

Subjects who reported at least 3 symptoms at 2.5 years after first infection had significantly more post-COVID syndromes at 5–6 months than subjects who were symptom-free (*p* = 0.0487) and also significantly more often than subjects who reported only 1–2 symptoms (“low” score) at 2.5 years after first infection (*p* = 0.0124) (Table 4).

## 4. Discussion

The clinical course of SARS-CoV-2 infection can be very variable [3,23,24], and is frequently followed by post-COVID syndromes even in non-hospitalized convalescents with an asymptomatic to moderate course of COVID-19 [7,11]. Current literature suggests that patients with severe COVID-19 develop a potent humoral immune response with high antibody levels against SARS-CoV-2 [23,25].

Here, we performed a long-term follow-up of 110 individuals 2.5 years after asymptomatic to moderate SARS-CoV-2 infection according to WHO criteria [18]. Vaccination behavior, persistent complaints or even ongoing post-COVID syndrome as well as reinfections were self-assessed and questionnaire-based. These data were considered in the context of humoral and cellular immune responses to SARS-CoV-2 that were assessed in a previous publication on a convalescent cohort [15,16,17,19].

Among the symptoms reported at the time of the survey, fatigue, difficulty concentrating, and reduced performance were the most frequently reported symptoms in our group. This is in line with the results of a meta-analysis involving 81 different studies [26]. We observed that persistent post-COVID symptoms occurred in approximately 40% of individuals who recovered from their first infection, which is in line with the results of another study, in which at least one post-infectious symptom was still present in 40.2% of convalescents six months after a positive PCR test [27]. However, in terms of severity, only a minority (15%) of participants in our study reported severe symptoms. In addition, we observed a trend towards elevated anti-spike IgG antibody levels 5–6 weeks after the positive PCR in the participants with long-term symptoms 2.5 years after infection. Regarding the association between higher antibody titers and long-term COVID-19 symptoms, there are conflicting results in this area: data from studies by Horton et al., Peghin et al., and van Elslande et al. report elevated SARS-CoV-2-specific anti-spike and anti-nucleocapsid antibody levels in patients with post-COVID syndrome assessed up to 6 months after infection, while other studies, such as those by García-Abellán et al., show controversial findings with a weak anti-SARS-CoV-2 antibody response associated with long-COVID-19 [17,25,27,28,29,30]. Conflicting results in the literature can be attributed to several factors, including heterogeneous study populations, timing of antibody measurements, and diverse antibody types, as well as methodological variations and sample size limitations. Regarding persistent symptoms, several studies have shown that persistent myalgias (muscle and joint pain) are very common (>50%) after COVID-19 [31,32,33,34]. Patients who were severely ill with COVID-19 (presenting with acute respiratory distress syndrome) were significantly more likely to be affected by this symptom [33,35]. Our results support these findings: 23% of convalescents in our study reported persistent myalgias, and these were associated with significantly higher anti-nucleocapsid and anti-spike IgG antibody levels after the first infection. In fact, even significantly higher antibody levels independent of the antibody class were found in individuals with severity scores of “severe” compared with individuals who had no symptoms at all. It should be noted, however, that chronic myalgias are not specific to SARS-CoV-2 and can occur after many other viral diseases [36,37,38].

To date, few data are available on the association between T-cell responses and COVID-19 symptoms, both acute and long-term [39]. Here, we used the previously reported data on SARS-CoV-2-specific and cross-reactive T-cell responses against HLA class I and HLA-DR-restricted SARS-CoV-2 T-cell epitopes for a corresponding correlative study [17]. Unlike humoral immune responses, the intensity of T-cell responses was not found to correlate with symptom severity in most cases and even decreased in individuals with more severe symptoms [40]. In our study, the presence of symptom fatigue was significantly associated with a lower T-cell response. However, further studies are needed to better understand potential associations.

In the literature, reinfection rates between 3–12% were described [41,42,43]: Guedes et al. [42] investigated the rate of reinfection in a cohort of healthcare workers over a span of 2 years during the COVID-19 pandemic. They identified reinfection with SARS-CoV-2 in 5% of symptomatic cases. Notably, the majority of reinfections occurred during the Omicron variant period, marking a significant increase in the SARS-CoV-2 reinfection rate compared to before and during the Omicron variant era (0.8% vs. 4.3%). In our study, a reinfection rate of 47% was observed and coincided temporally with the Omicron wave [22]. The Omicron variant exhibited a higher rate of transmission compared to the Gamma and Delta variants, as evidenced by the progressively shorter transition periods [42]. In addition, possible reasons for the higher reinfection rate in our study could be the sample size, the behavior of convalescents, such as adherence to protective measures (e.g., wearing masks, social distancing, vaccination), and declining immunity since the first infection and last vaccination.

Furthermore, we observed significantly lower anti-spike and anti-nucleocapsid antibody levels 5–6 weeks after first-time positive PCR test for SARS-CoV-2 in patients who subsequently had a reinfection, which may help to identify individuals at increased risk for reinfection, as anti-nucleocapsid antibodies are not induced by EMA-approved vaccines. Moreover, faster recovery after acute COVID-19 is associated with higher anti-nucleocapsid antibody levels in the first week after diagnosis, further highlighting its importance [44]. Finally, our data also confirm the findings of the study by Harvey et al. and others, in which higher antibody levels after the first infection protect against reinfection [45,46,47,48].

In our study, 92% of convalescents were vaccinated at least once, indicating high utilization of the vaccination service. Interestingly, 3% of study participants reported long-term sequelae after vaccination; however, the present study did not distinguish the type or severity of these sequelae, and the presence of long-term sequelae after vaccination was self-assessed. According to the Paul Ehrlich Institute [49], the rate of suspected cases of serious adverse events after SARS-CoV-2 vaccination in Germany is 0.29 per 1000 vaccinations (all vaccines), with these adverse events usually occurring shortly after vaccination. In individual cases, vaccine complications may persist over a longer period of time [50,51,52]. Therefore, the relative high percentages reported in this study should be interpreted cautiously, as these are subjective statements by the participants and not objective clinical parameters. It should also be emphasized that the self-assessment of symptoms was conducted using questionnaires; therefore, the interpretation of the results should be performed with caution, as this may introduce subjectivity and carry a potential impact of recall error [53,54]. The non-specific nature of the reported symptoms and the difficulty of attributing them exclusively to COVID-19 has to be considered when interpreting the data in this study. Therefore, it would be important for further research to take differential diagnoses into account, as this is highly needed to distinguish COVID-19-related symptoms from those of other disorders. Further limitations of our study include the missing control group in our study. The inclusion of a control group would be essential for further studies, which aim to confirm the beforementioned conclusions about the association between long-term symptoms and immune responses measured at initial infection with SARS-CoV-2.

In summary, this study provides further insight into the relationship between the SARS-CoV-2-specific humoral and cellular immune response and the persistency of post-COVID syndrome as well as reinfections: elevated SARS-CoV-2-specific antibody levels were found early after infection in convalescents with ongoing post-infectious symptoms approximately 2.5 years after the first infection. High anti-spike IgG antibody levels were associated with a lower probability of reinfection.

## Figures and Tables

**Table 1:** Presence and distribution of symptom severities 2.5 years after first infection; n: number of donors. %: percentage of all donors.

**Presence of symptoms 2.5 years after first infection [n (%)]**					
0 symptoms regardless of the severity			38 (35)		
≥1 symptom regardless of the severity			72 (65)		
**Maximum severity achieved in patients for any of assessed symptoms [n (%)]**					
None			38 (35)		
Mild			28 (25)		
Noderate			27 (25)		
Severe			17 (15)		
**Symptom**	**n**	**Patients with symptoms** **[n (%)]**	**Severity [n (%)]**		
			**Mild**	**Moderate**	**Severe**
**Breathlessness**	110	33 (30)	17 (15)	16 (15)	0 (0)
**Fatigue**	110	46 (42)	27 (25)	9 (8)	10 (9)
**Difficulty concentrating**	110	37 (34)	19 (17)	13 (12)	5 (5)
**Reduced performance**	110	37 (34)	18 (16)	16 (15)	3 (3)
**Sleep disorders**	110	31 (28)	19 (17)	7 (6)	5 (5)
**Depression**	110	24 (22)	13 (12)	10 (9)	1 (1)
**Loss of taste/smell**	110	14 (13)	9 (8)	2 (2)	3 (3)
**Muscle/joint pain**	110	25 (23)	14 (13)	6 (5)	5 (5)
**Hearing loss/disorder**	110	12 (11)	9 (8)	1 (1)	2 (2)
**Headache**	110	22 (20)	14 (13)	7 (6)	1 (1)
**Menstruation disorders**	55	1 (2)	0 (0)	0 (0)	1 (2)

**Table 2:** Number of vaccinations received after first infection, long-term symptoms after vaccination, distribution of used vaccinations for the first, second, third, and fourth vaccination, and n: number of donors. %: percentage of donors.

**Number of vaccinations after first infection (n [%])**						
**0 vaccinations**			4 (4)			
**1 vaccination**			7 (6)			
**2 vaccinations**			47 (43)			
**3 vaccinations**			45 (41)			
**4 vaccinations**			7 (6)			
**Long-term symptoms after vaccination (n [%])**						
**Self-assessed presence of symptoms**			3 (3)			
**Distribution of used vaccines (n [%])**						
	**Tozinameran** **[n (%)]**	**Elasomeran** **[n (%)]**	**AZD1222** **[n (%)]**	**Ad26.COV2.S** **[n (%)]**	**Other** **[n (%)]**	**n**
**First** **vaccine**	65 (61)	16 (15)	20 (19)	4 (4)	1 (1)	106
**Second vaccine**	73 (74)	22 (22)	3 (3)	0 (0)	1 (1)	99
**Third** **vaccine**	35 (67)	15 (29)	1 (1)	0 (0)	1 (1)	52
**Fourth vaccine**	5 (71)	2 (29)	0 (0)	0 (0)	0 (0)	7
**Sum of used vaccines**	178 (67)	55 (21)	24 (9)	4 (2)	3 (1)	n_total_ = 264

**Table 3:** Reinfection rate after first infection; n: number of donors. %: percentage of donors.

**Reinfection Rate after First Infection [n (%)]**	
No reinfection	59 (53)
≥2 infections	51 (47)
3 infections	4 (4)

**Table 4:** Post-COVID syndrome after 5–6 months and long-term symptoms at 2.5 years after first infection; *p*-values were calculated by Chi^2^-test. *p*, *p*-value; n: number of donors; %: percentage of donors.

**Score for Long-Term** **Symptoms at 2.5 Years**	**No Post-COVID** **Syndrome at 5–6 Months**	**Post-COVID** **Syndrome at 5–6 Months**	***p*-Value**
None (0 symptoms) [n (%)]	6 (75)	2 (25)	0.7189
Low (1–2 symptoms) [n (%)]	9 (82)	2 (18)	
None (0 symptoms) [n (%)]	6 (75)	2 (25)	0.0487
High (3–10 symptoms) [n (%)]	4 (31)	9 (69)	
Low (1–2 symptoms) [n (%)]	9 (82)	2 (18)	0.0124
High (3–10 symptoms) [n (%)]	4 (31)	9 (69)	

## Data Availability

The data presented in this study are available upon request from the corresponding author.

## Supplementary Materials

The following supporting information can be downloaded at: https://www.mdpi.com/article/10.3390/v15102100/s1, Table S1: donor characteristics; Table S2: symptom severity at reinfection and course of second infection compared to first infection. Figure S1: Long-term symptoms and antibody response in SARS-CoV-2 convalescents; Figure S2: Severity of “muscle and joint pain” and antibody response in SARS-CoV-2 convalescents; Figure S3: Reinfection and T-cell response in SARS-CoV-2 convalescents.
