# Supplementary material for: Long-Term Follow-Up of COVID-19 Convalescents—Immune Response Associated with Reinfection Rate and Symptoms
Source: Viruses. 2023 Oct 17;15(10):2100. doi: 10.3390/v15102100 (PMC10611319; doi:10.3390/v15102100)
Supplement: Supplementary file 1 [file viruses-15-02100-s001.zip › viruses-2628612-supplementary.pdf]

Table S1. Donor characteristics; n: number of donors. %: percentage of donors. PCR: polymerase chain reaction. d: days.

	COVID-19 convalescent donors
number of donors	110
age (years)	
range	20 - 75
median	49
sex (n (%))	
female	57 (52)
male	53 (48)
time PCR to sample collection (d)	
range	16 - 59
median	41

Table S2. Symptom severity at reinfection and course of second infection compared to first infection; n: number of donors. %: percentage of donors.

**symptom severity at reinfection [n (%)]**					
	no [n (%)]	mild [n (%)]	moderate [n (%)]	severe [n (%)]	
symptoms at second infection (n = 51/110)	1 (2)	1 (2)	18 (35)	31 (61)	
symptoms at third infection (n = 4/51)	0 (0)	0 (0)	2 (50)	2 (50)	
**course of second infection compared to first infection [n (%)]**					
n = 52	no [n (%)]	same [n (%)]	mild [n (%)]	moderate [n (%)]	severe [n (%)]
course second compared to first infection worse?	21 (40)	12 (23)	9 (17)	4 (8)	6 (12)

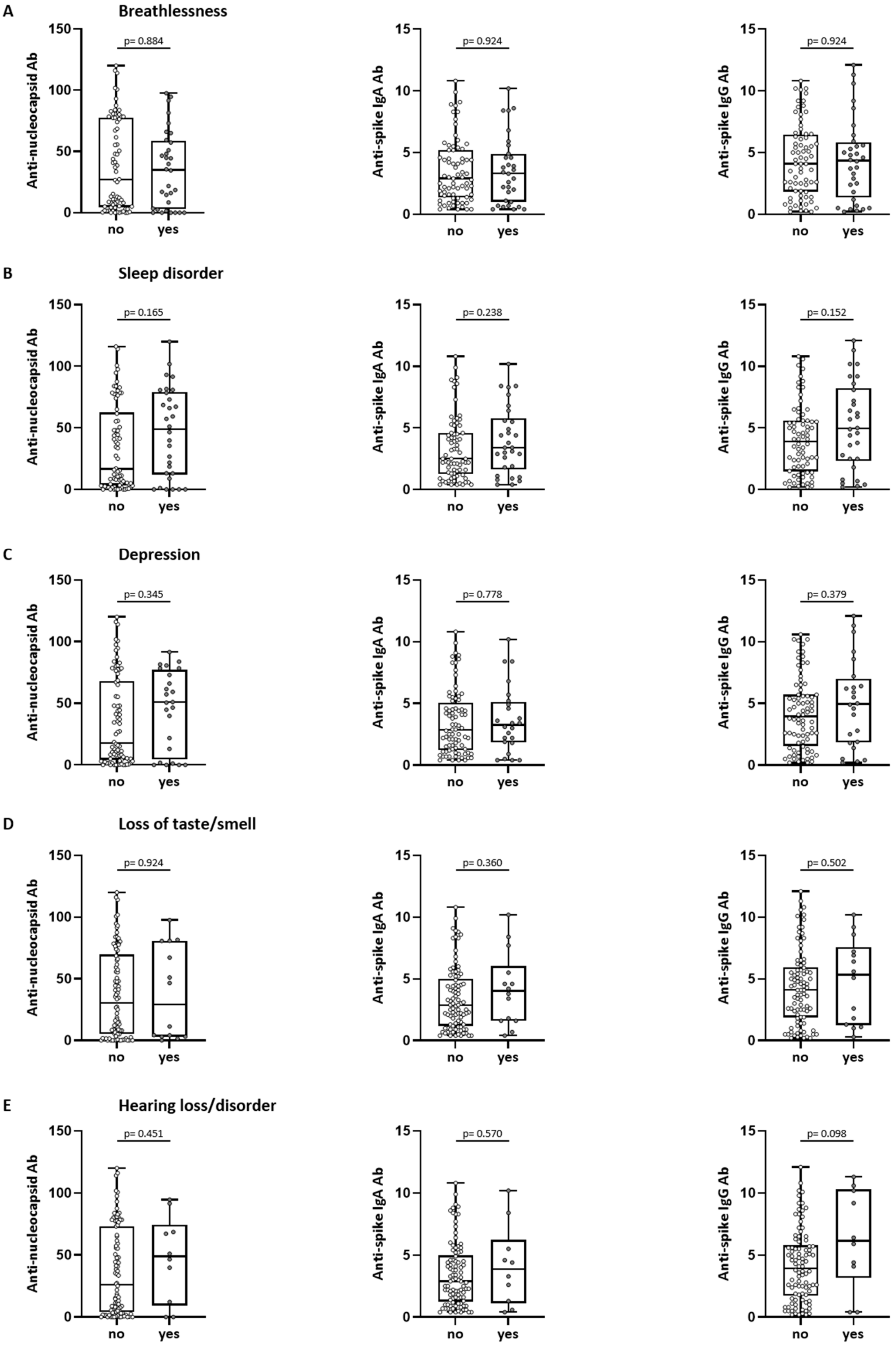

Figure S1. Long-term symptoms and antibody response in SARS-CoV-2 convalescents. Anti-nucleocapsid antibody (Ab) (left) and anti-spike Ab levels (IgA middle, IgG right) were assessed in convalescent donors (n = 110) 5–6 weeks after positive PCR at first infection. Convalescents were grouped into "yes" (reported symptom)

or “no” (no perception of symptom) about 2.5 years after first infection. Levels of anti-spike Ab are shown as ratio above threshold value. Levels of anti-nucleocapsid Ab are shown as an index value. Data are presented as box plots with 25th and 75th percentiles and min/max whiskers. *p*-values were calculated by Mann-Whitney-U test. *p*, *p*-value; Ab, antibody.

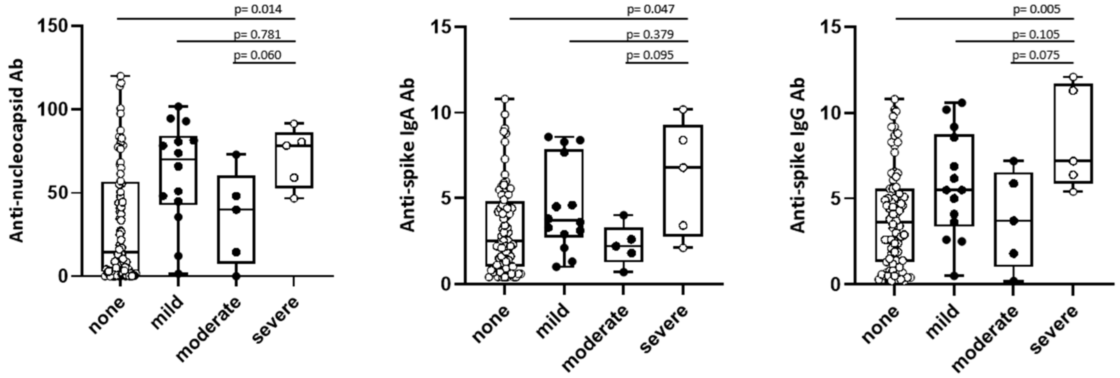

Figure S2. Severity of “muscle and joint pain” in association with antibody response in SARS-CoV-2 convalescents. Anti-nucleocapsid antibody (Ab) (left) and anti-spike Ab levels (IgA middle, IgG right) were assessed in convalescent donors (n = 110) 5-6 weeks after positive PCR at first infection. Convalescents were grouped according to symptom severity 2.5 years after first infection. Existence and severity (none, mild, moderate, severe) of muscle and joint pain was assessed by questionnaire. Levels of anti-spike Ab are shown as ratio above threshold value. Levels of anti-nucleocapsid Ab are shown as an index value. Data are presented as box plots with 25th and 75th percentiles and min/max whiskers. *p*-values were calculated by Mann-Whitney-U test. *p*, *p*-value; Ab, antibody.

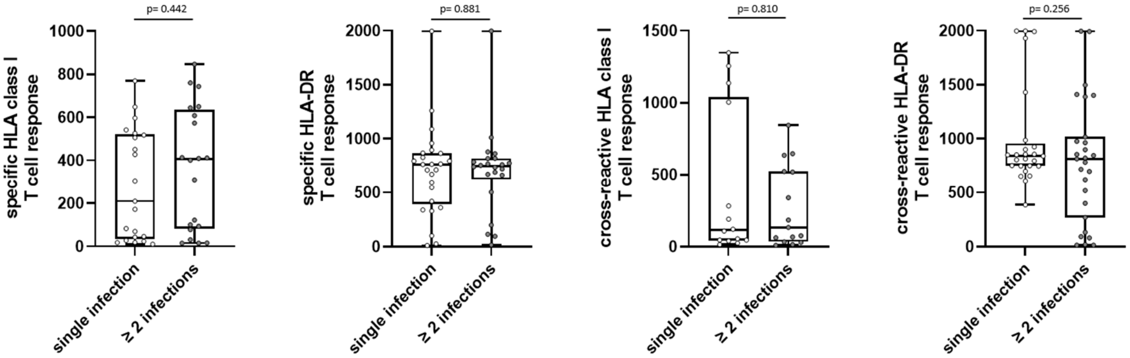

Figure S3. Reinfection and T cell response in SARS-CoV-2 convalescents. Specific HLA class I (left)/HLA-DR (second from left) T cell response as well as cross-reactive HLA class I (second from right)/HLA-DR (right) T cell response were assessed in convalescent donors (n = 110) 5-6 weeks after positive PCR at first infection. Convalescents were grouped into “single infection” or “≥2 infections”. Intensity levels of T cell responses are shown as normalized mean spot counts to the respective negative control. Data are presented as box plots with 25th and 75th percentiles and min/max whiskers. *p*-values were calculated by Mann-Whitney-U test. *p*, *p*-value.
